# A UPLC/DAD method for simultaneous determination of empagliflozin and three related substances in spiked human plasma

**DOI:** 10.1186/s13065-019-0604-9

**Published:** 2019-07-09

**Authors:** Mokhtar M. Mabrouk, Suzan M. Soliman, Heba M. El-Agizy, Fotouh R. Mansour

**Affiliations:** 1 Department of Pharmaceutical Analytical Chemistry, Faculty of Pharmacy, Tanta University, Elgeish Street, Medical Campus, Tanta, 31111 Egypt; 2 Pharmaceutical Services Center, Faculty of Pharmacy, Tanta University, Tanta, 31111 Egypt; 3 National Organization for Drug Control and Research (NODCAR), Giza, 12511 Egypt

**Keywords:** Empagliflozin, Related substances, UPLC, Spiked human plasma, THF protein precipitation

## Abstract

A simple, rapid and sensitive ultrahigh performance liquid chromatographic method was developed for the determination of the anti-diabetic drug: empagliflozin (EMPA) and three related substances in spiked human plasma, using dapagliflozin (DAPA) as an internal standard and tetrahydrofuran as a plasma protein precipitating agent. The chromatographic separation was achieved on an Acquity “UPLC^®^ BEH” C18 column (50 mm × 2.1 mm i.d, 1.7 µm particle size), and a mobile phase consisting of aqueous trifluoroacetic acid (0.1%, pH 2.5): acetonitrile (60:40, v/v) at a flow rate of 0.5 mL/min. Upon using the UPLC system, the run time could be reduced to less than 1.2 min, and the solvents consumption decreased to 0.36 mL of acetonitrile per run. The response was linear over a concentration range of 50–700 ng/mL and 40–200 ng/mL (r^2^ = 0.9994–0.9999) with lower limits of detection and quantification (LOD/LOQ) of 15/50, 11.5/40, 12/40 and 12.5/40 ng/mL for EMPA and the three related substances, respectively. Good accuracy was obtained with mean percentage recoveries ≥ 96.97% for the studied compounds. The method was validated according to the ICH guidelines and was found suitable for routine analysis of EMPA and its related substances in human plasma.

**Electronic supplementary material:**

The online version of this article (10.1186/s13065-019-0604-9) contains supplementary material, which is available to authorized users.

## Introduction

Empagliflozin (EMPA) is a new oral antidiabetic drug, prescribed mainly for the treatment of type 2 diabetes. EMPA works by selective inhibition of sodium glucose cotransporter-2 (SGLT-2) [1]. The new pharmacological class of SGLT2-inhibitors lower blood glucose levels by targeting the kidney, reducing renal glucose reabsorption, and increasing urinary glucose elimination [2]. Determination of EMPA in bulk and/or pharmaceutical dosage forms as well as in human plasma has been reported in a few methods using spectrophotometric techniques [3, 4] and liquid chromatography [5–21]. Related substances are either produced as impurities from the manufacturing process, degradation products from improper storage or as metabolites that are either active, inactive or even toxic [22].

**Fig. 1:**
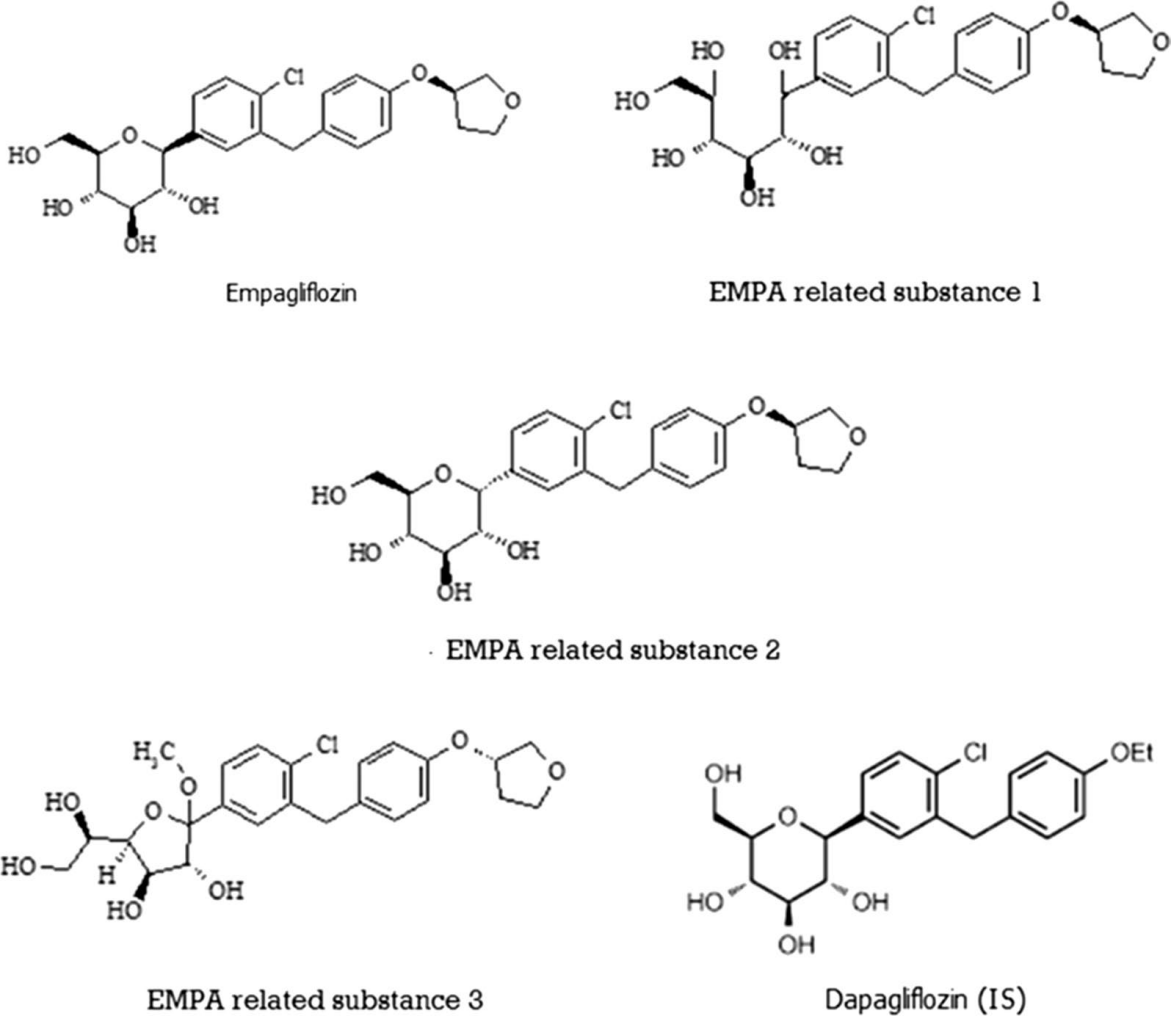
Chemical structures of empagliflozin (EMPA), its three related substances (ERS1–3) and dapagliflozin (DAPA) (IS)

The present study is interested in analysis of EMPA and three EMPA related substances (ERSs) that result from ring opening (ERS1), isomerization (ERS2) and alkylation/ring size reduction (ERS3). Figure 1 shows the chemical structure of EMPA and the related substances (ERS1–3). In spite of the many analytical techniques used for analysis of EMPA either alone [5–9] or in combination with other co-formulated drugs [10–18] and the available pharmacokinetic studies in literature [19–21], there are no analytical methods available with full details regarding plasma extraction and determination of EMPA and its three related substances in plasma samples to evaluate the pharmacokinetic parameters of the studied compounds. To the best of our knowledge, the present analysis is the first UPLC method carried out for the simultaneous determination of EMPA and these related substances in human plasma. The aim of this work is to develop a simple, fast, sensitive and fully validated UPLC/DAD method for separation and quantitation of EMPA and the three related substances using dapagliflozin (DAPA) as an internal standard.

## Experimental

### Instruments

Chromatographic separation was carried out on an ACQUITY UPLC system (Waters Corp., Milford, MA, USA). The UPLC was equipped with a quaternary solvent manager SN C15QSM334A ver 1.65.287 and a temperature-controlled autosampler SN C155DI089G ver 1.65.375, coupled to a DAD detector SN C15UPL193A ver 1.65.6229 and connected to Waters Empower 2 software.

### Materials and reagents

Empagliflozin standard sample and its three related substances were kindly provided by Boehringer Ingelheim (Ingelheim, Germany), with 99.98% purity for the drug, based on the company analysis certificate. DAPA propanediol monohydrate was kindly supplied by AstraZeneca (Giza, Egypt) and was used as an internal standard (IS). DAPA purity was found to be 99.59%. Acetonitrile and methanol (HPLC grade) were purchased from J.T.Baker (Phillipsburg, NJ, USA) and Fischer Chemical (Loughborough, UK), respectively. Tetrahydrofuran (THF) and trifluoroacetic acid were purchased from Sigma-Aldrich (St. Louis, MO, USA) and Carlo Erba reagents (Peypin, France), respectively. Deionized water was obtained from a Milli-Q water purification system (Millipore, USA). Human plasma samples were kindly supplied from Vacsera National Blood Bank, Egypt, frozen until use after gentle thawing.

### Chromatographic conditions

**Fig. 2:**
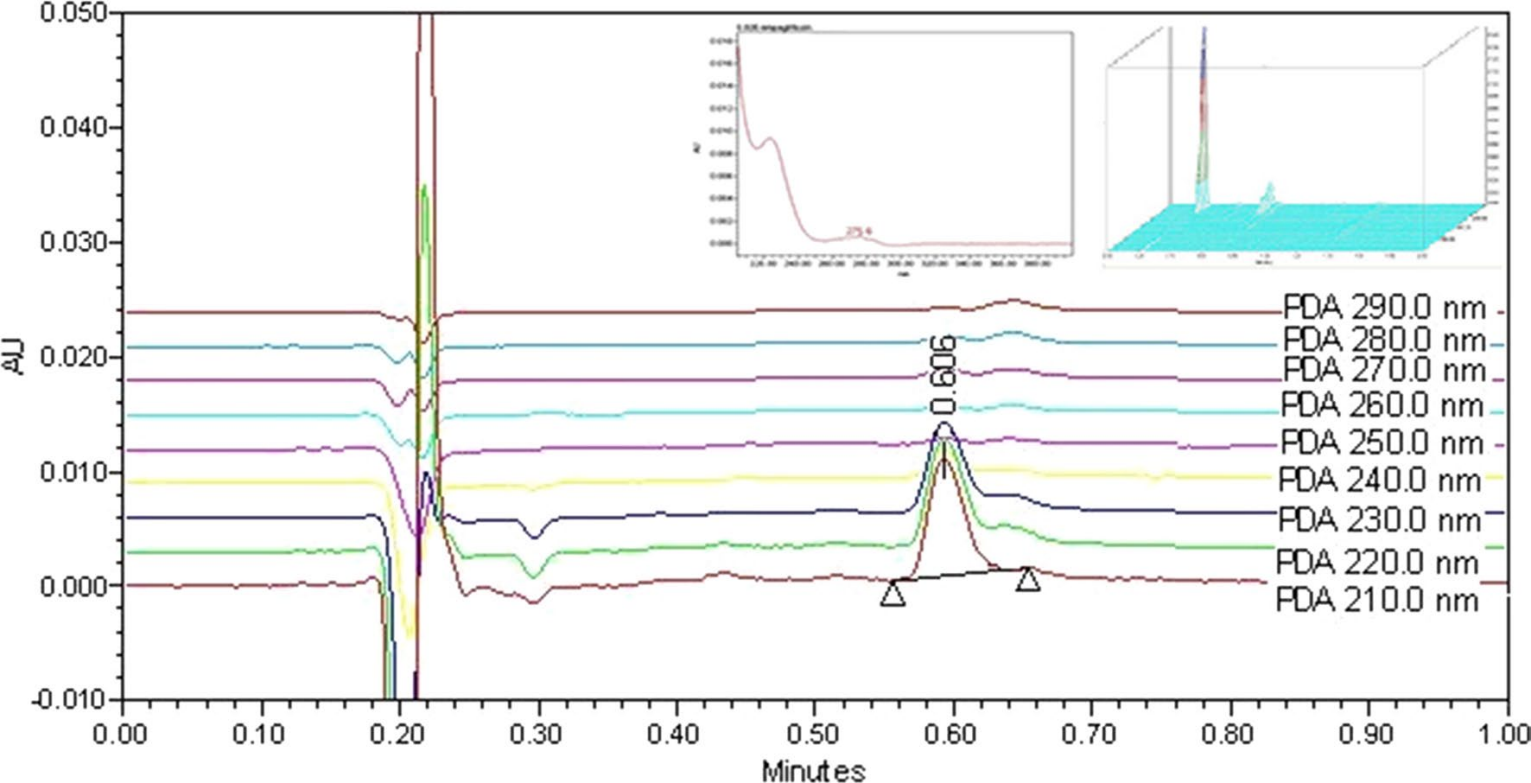
An overlay of EMPA chromatograms at different wavelengths (210–290 nm)

Separation of the analytes was performed on an Acquity UPLC^®^ BEH C18 column (50 mm × 2.1 mm i.d, 1.7 μm, Waters Corp., USA); the column oven temperature was maintained at 50 °C. The samples were eluted with an isocratic mobile phase consisting of acetonitrile-aqueous 0.1% trifluoroacetic acid pH 2.5, (40:60, *v/v*), pH was adjusted to pH 2.5 with glacial acetic acid. The flow rate was 0.5 mL/min and the injection volume was 5 μL. The response of the photodiode array detector (DAD) over the range 200–400 nm was studied. Detection and quantitation of the analytes were performed at optimum intensity of λ 210 nm (Fig. 2).

### Preparation of standard solutions

Stock solutions of EMPA (100 µg/mL), each of the related substances (400 µg/mL) and DAPA (1000 µg/mL) were prepared in the mobile phase and stored at 4 °C until use. The working solutions were freshly prepared by the appropriate dilution of the stock solutions with the mobile phase to obtain 5 µg/mL of DAPA in each solution as an internal standard with EMPA in the range 50–700 ng/mL and ERS in the range 40–200 ng/mL. All solutions were stored at 2–8 °C and brought to room temperature before use.

### Plasma samples preparation

After thawing the samples at room temperature, 10 µL of the IS solution was added in each tube and the sample was vortexed for 30 s. After that, 1 mL of THF was added to an aliquot containing plasma, EMPA, ERS1, ERS2, ERS3 and IS. The mixture was mixed again, vortexed and then centrifuged at 4000 rpm for 5 min; the supernatant was filtered through a 0.2 μm syringe filter and carefully transferred into a UPLC vial and 5 µL were injected into the UPLC system.

Calibration curves were constructed from a blank sample (a plasma sample without the IS), a zero sample (a plasma spiked with the IS) and non-zero samples covering the total range of 50–700 ng/mL and 40–200 ng/mL for EMPA and its three related substances, respectively; including the lower limit of quantification (LLOQ). The concentrations of the drug and its related substances were determined using the corresponding regression equations. The samples were stored in a freezer at − 20 °C until analysis, and then allowed to thaw at 25 °C before processing. The plasma samples were centrifuged at 4000 rpm for 5 min, for each concentration.

### Stability of EMPA in human plasma

The acceptable stability of the analytes in spiked plasma during sample storage and during processing conditions was investigated by analyzing the drug at five levels: lower limit of quantitation (LLOQ, 50 ng/mL), low quality control (LQC, 100 ng/mL), medium quality control (MQC, 300 ng/mL), high quality control (HQC, 500 ng/mL) and upper limit of quantitation (ULOQ, 700 ng/mL) and the results were compared with that of zero cycles. The short-term stability (bench-top stability) was determined after sample storage at room temperature for 24 h, freeze–thaw stability was determined over three freeze–thaw cycles within 3 days. In each cycle, the frozen plasma samples were thawed at room temperature for 2 h and refrozen for 24 h. The long-term stability was determined at the same five QC levels (50, 100, 300, 500 and 700 ng/mL) after sample storage at − 20 °C for 30 days. The concentration of EMPA after each storage period was related to the initial concentration at zero cycle (samples that were freshly prepared and processed immediately). The samples were considered stable if the deviation (expressed as percentage bias) from the zero cycle was within 15%.

## Results and discussion

Empagliflozin is orally active with a 78% bioavailability after a single oral dose [23]. The peak plasma concentrations of EMPA is reached at 1.5 h post-dose. EMPA plasma concentrations decline in a biphasic manner with a rapid distribution phase and a relatively slow terminal phase. The steady state mean plasma AUC and C_max_ are 843.2 ng h/mL and 116.8 ng/mL, respectively, with 10 mg EMPA once daily treatment [23].

### Method development and optimization

Since EMPA is highly bound to plasma proteins [24], protein precipitation by a suitable precipitating agent is crucial to denature the plasma proteins and liberate the free drug. Three different protein precipitants were tried including acetonitrile, methanol and tetrahydrofuran in 1:1 ratio (plasma:precipitating agent, v/v). The peak intensity of EMPA and the baseline drift were compared. Tetrahydrofuran was found to give the highest peak intensity and the most stable baseline.

**Fig. 3:**
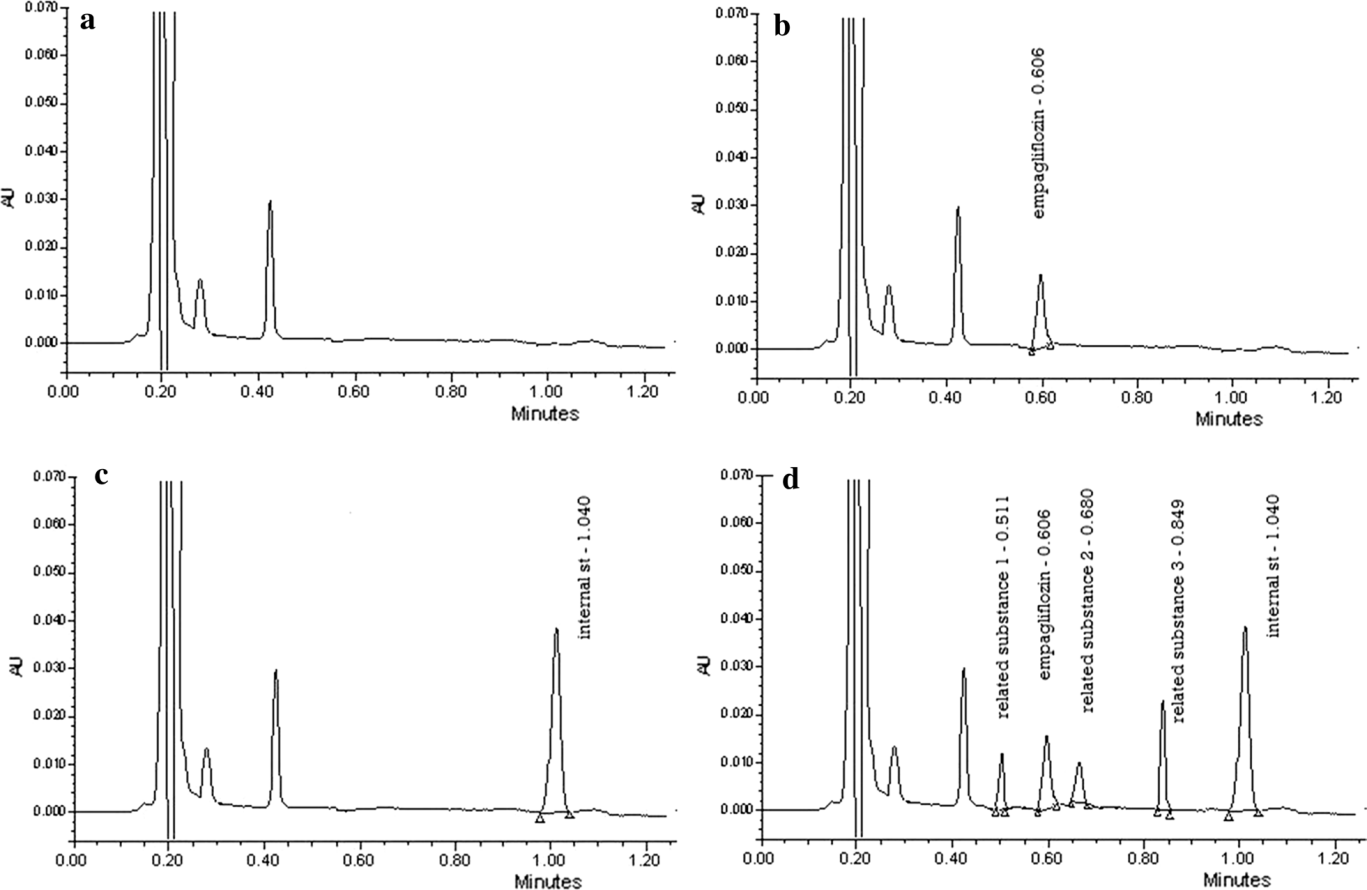
Chromatograms of **a** blank plasma, **b** plasma spiked with 700 ng/mL EMPA, **c** plasma spiked with 5 µg/mL I.S, **d** plasma spiked with 700 ng/mL EMPA, 200 ng/mL of each related substance and 5 µg/mL IS

To correct for sample loss during preparation, an internal standard was added. Several drugs were tested, DAPA which is structurally related to EMPA drug and possess an ethyl group in place of the oxolane moiety, was selected as the appropriate IS, giving an acceptable retention time with a symmetrical peak shape (Fig. 3).

Increasing the sensitivity and the selectivity of the developed method have been taken into consideration during method development by optimizing detection wavelength, pH of the aqueous portion of the mobile phase, percentage of acetonitrile in the mobile phase, the diluting solvent and the column temperature before analysis.

The effect of different pH values in the mobile phase were carefully studied at pH 2.5, 3.5 and 4.5. Although the elution of EMPA and DAPA peaks seemed to happen a bit earlier in pH 3.5 and 4.5 than in case of pH 2.5 (Additional file 1: Fig. S1) but the peak areas decreased with increasing pH. A mobile phase pH of 2.5 was selected to attain a chromatogram with high peak areas, optimum resolution, and a less drifty baseline.

Percentages of acetonitrile in the mobile phase were studied using three different ratios (20:80, 30:70 and 40:60, *v/v* of acetonitrile-aqueous 0.1% trifluoroacetic acid pH 2.5). The ratio 40:60 with a flow rate of 0.5 mL/min achieved the highest peak areas and the fastest elution as well (Additional file 1: Fig. S2).

The solvent, used for dilution, had a great influence on peaks’ shape, area and retention times. Upon using pure methanol as a diluting solvent, the peaks of drug and IS were neither sharp nor symmetric and a huge solvent peak were observed in the chromatogram, while on using water–methanol (50:50, v/v) the peaks of the analytes were improved in shape but a huge solvent peak was observed. This problem was diminished by using the mobile phase as a diluting solvent (Additional file 1: Fig. S3).

Column temperature was assessed by using three different column oven temperatures: 30, 40 and 50 °C, a very poor peak shape and area were obtained at low temperatures (≤ 40 °C), increasing column oven temperature to 50 °C greatly enhanced both shape and intensity of the peaks (Additional file 1: Fig. S4). The optimum separation was achieved using an isocratic mobile phase consisting of acetonitrile: trifluoroacetic acid (0.1%, pH 2.5) (40:60, *v/v*), at a flow rate of 0.5 mL/min and an injection volume of 5 μL and a column temperature of 50 °C.

System suitability was assessed by calculating capacity factor (− k′), selectivity (α), resolution (R_s_), tailing factor (T), and number of theoretical plates (N), where the system was found to be suitable for the intended purpose under the specified conditions (Table 1).

**Table 1:** System suitability parameters by the proposed UPLC method

Parameters	ERS1	EMPA	ERS2	ERS3	IS	Recommended value^a^
Retention time	0.511	0.606	0.68	0.849	1.040	–
− k′	1.55	2.12	2.49	3.26	4.39	1–10 acceptable
α	–	1.37	1.17	1.31	1.76	> 1
R_s_	–	2.15	2.01	5.51	7.81	> 2
T	0.95	1.01	1.05	0.98	1.3	≤ 2 T = 1 for a typical symmetric peak
N	12,890	3920	4023	13,205	4527	2000–10,000 increases with efficiency of the separation

^a^Values defined by FDA Center of Drug Evaluation and Research’s reviewer guidance on validation of chromatographic methods [25]

### Method validation

The validity of the method was assessed for linearity, specificity, accuracy, repeatability, recovery and precision according to the ICH bio-analytical method validation guidelines [25].

A calibration curve was constructed by plotting the ratio of peak areas (drug/IS) against concentration of the analyte in the plasma. Standard calibration curves exhibited good linearity over the concentration range 50–700 ng/mL for EMPA and 40–200 ng/mL for the three related substances, with good correlation coefficient and regression data as shown in Table 2.

**Table 2:** Validation parameters of the proposed UPLC method for the determination of EMPA and its three related substances in spiked human plasma

Parameter	EMPA in spiked human plasma	ERS1	ERS2	ERS3
Range (ng/mL)	50–700	40–200	40–200	40–200
n	8	5	5	5
LOD (ng/mL)^a^	15	11.5	12	12.5
LLOQ (ng/mL)^b^	50	40	40	40
Accuracy				
Intra-day^c^	97.09	97.86	99.28	96.97
Inter-day^d^	98.18	99.47	98.20	99.67
Precision ± %RSD				
Intra-day^c^	± 6.38	± 7.26	± 7.94	± 7.34
Inter-day^d^	± 6.63	± 7.93	± 6.60	± 7.42
Linearity				
Slope	0.0003	0.0002	0.0005	0.0007
Standard deviation of slope	5.7 × 10^−6^	5.8 × 10^−6^	7.6 × 10^−6^	6.8 × 10^−6^
Intercept	− 0.0016	− 0.0008	− 0.0004	− 0.0032
Standard deviation of intercept	0.00001	0.00002	0.00001	0.00007
Correlation coefficient (r)	0.9999	0.9994	0.9999	0.9997

^a^LOD is the lowest detectable amount of the selected drug that could be detected

^b^LLOQ is the lowest concentration of the analyte that can be measured accurately under the proposed experimental condition

^c^The intra-day study was performed for five concentrations for EMPA and its related substances (ERS 1–3), repeated three times within the day

^d^The inter-day study was performed for five concentrations for EMPA and its related substance (ERS 1–3), repeated three times in 3 successive days

The limit of detection (LOD) is defined as the lowest detectable amount of the selected drug that could be detected, while lower limit of quantification (LLOQ) is the lowest concentration of the analyte that can be measured accurately and precisely under the proposed experimental conditions. LLOQ should meet the acceptable criteria (precision = ± 20% and the accuracy within 80–120%). The values of the LLOQ were 50 ng/mL and 40 ng/mL for EMPA and the three ERSs, respectively (Table 2).

Accuracy and precision were evaluated by analysis of quality control samples in the range 50–700 ng/mL for EMPA and 40–200 ng/mL for EMPA related substances, using three determinations per concentration on 3 consecutive days. The accuracy was expressed as %recovery while the precision was monitored by the %RSD. Table 3 summarizes the results of accuracy and precision for the intra- and inter-day analysis of the analytes in plasma. The proposed UPLC method was able to determine different concentrations of EMPA over the working concentration range with %RSD less than 15%.

**Table 3:** Recovery and precision of EMPA and its related substances

Compound	Concentration added (ng/mL)	Intra-day% (n = 5)			Inter-day% (n = 5)		
		Concentration found (ng/mL)	Recovery % (n = 5)	%RSD	Concentration found (ng/mL)	Recovery % (n = 5)	%RSD
EMPA	50 (LLOQ)	46.21	92.42	13.8	47.15	94.30	11.1
	100 (LQC)	98.60	98.60	4.34	96.22	96.22	6.38
	300 (MQC)	293.79	97.93	5.90	301.7	100.6	9.45
	500 (HQC)	501.36	100.27	4.22	494.3	98.86	3.61
	700 (ULOQ)	673.54	96.22	3.67	706.5	100.9	2.59
Mean			97.09	6.38		98.18	6.63
ERS1	40 (LLOQ)	36.19	90.48	11.9	43.71	109.3	12.4
	80 (LQC)	84.48	105.6	6.76	75.98	94.98	9.11
	120 (MQC)	114.6	95.50	8.42	110.7	92.25	8.92
	160 (HQC)	154.9	96.84	3.90	156.2	97.63	4.15
	200 (ULOQ)	201.7	100.9	5.32	206.3	103.2	5.07
Mean			97.86	7.26		99.47	7.93
ERS2	40 (LLOQ)	36.68	91.70	13.2	35.82	89.55	14.3
	80 (LQC)	77.86	97.33	5.92	75.37	94.21	7.05
	120 (MQC)	125.5	104.6	7.09	118.6	98.83	2.64
	160 (HQC)	164.6	102.9	6.39	169.4	105.9	6.08
	200 (ULOQ)	199.7	99.85	7.11	204.9	102.5	2.94
Mean			99.28	7.94		98.20	6.60
ERS3	40 (LLOQ)	37.72	94.30	14.6	38.70	96.75	12.9
	80 (LQC)	81.68	102.1	9.63	79.11	98.89	10.3
	120 (MQC)	111.7	93.08	6.37	122.9	102.4	7.25
	160 (HQC)	154.9	97.34	4.08	164.3	102.7	3.69
	200 (ULOQ)	196.1	98.05	2.01	195.2	97.60	2.98
Mean			96.97	7.34		99.67	7.42

**Fig. 4:**
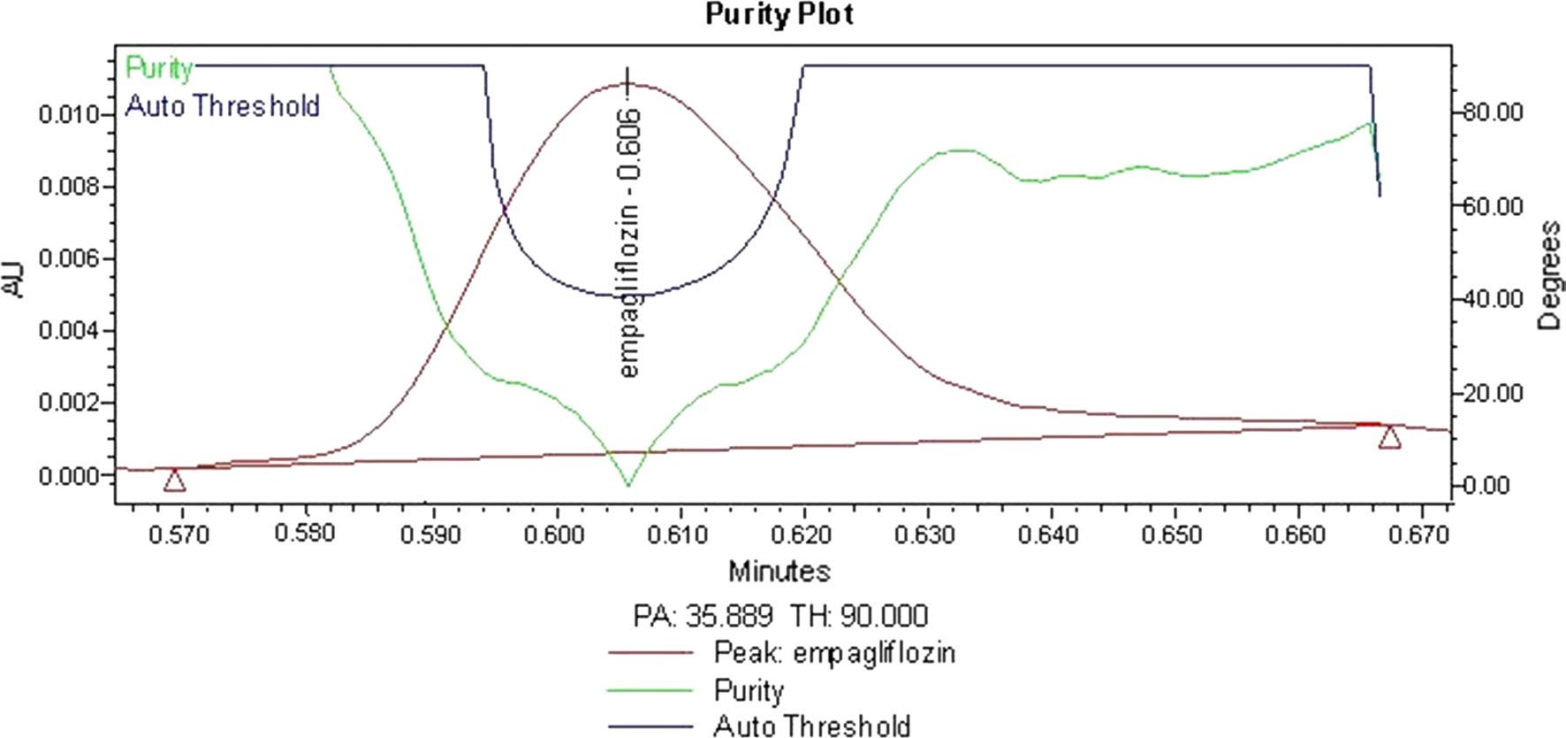
Purity plot of analyte peak demonstrates the homogeneity of the EMPA drug

The selectivity of the method was assessed by analyzing six different batches of human plasma to check for interference from endogenous substances. In addition, the peak purity of EMPA was checked by using a DAD detector. The calculated purity angle (35.89) was found to be less than the purity threshold (90) which demonstrates the homogeneity of the analyte peak and indicates that it had passed the peak purity test, as illustrated in Fig. 4.

Robustness of the method was assessed by undergoing minor changes in the experimental parameters such as, changing the volume of acetonitrile ± 2 mL, pH ± 0. 2, column temperature ± 2 °C and wavelength ± 2 nm. These minor changes that may take place during the experimental operation did not significantly affect the peak area values of the analytes.

The stability of the analyte in plasma was assessed at varying stability conditions. The samples were analyzed and the results were compared with that obtained with the corresponding freshly prepared and immediately processed samples. EMPA showed stability in spiked human plasma when stored at ambient temperature for at least 24 h, also when stored at − 20 °C for 1 month as long-term stability, and over three freeze–thaw cycles. The results indicate reliable stability during analysis and no stability-related problems during bio-analytical studies (Table 4).

**Table 4:** Stability of EMPA in human plasma

Parameters	Concentration (ng/mL)	%Recovery	%RSD	%RE
Short term At 25 °C, 24 h	50 (LLOQ)	95.37	13.5	− 4.63
	100 (LQC)	104.1	10.9	4.10
	300 (MQC)	93.71	9.62	− 6.29
	500 (HQC)	101.5	8.07	1.50
	700 (ULOQ)	99.01	4.39	− 0.99
Long term At − 20 °C, 30 days	50 (LLOQ)	90.63	11.2	− 9.37
	100 (LQC)	93.82	7.37	− 6.18
	300 (MQC)	95.13	5.97	− 4.87
	500 (HQC)	103.7	7.11	3.68
	700 (ULOQ)	108.4	8.94	8.42
Three freeze–thaw cycles thawed at 25 °C for 2 h, refrozen 24 h	50 (LLOQ)	92.07	12.9	− 7.93
	100 (LQC)	101.7	8.66	1.70
	300 (MQC)	91.91	10.1	− 8.09
	500 (HQC)	93.44	9.79	− 6.56
	700 (ULOQ)	97.12	4.64	− 2.88

## Conclusion

A novel, fast, sensitive, selective and fully validated UPLC/DAD method was developed for analysis of EMPA and its three related substances in spiked human plasma. Favorable advantages of the proposed UPLC method were found in significant reduction in elution time as well as simplicity of sample preparation and minimization of solvent consumption when compared with other reported methods. Moreover, THF is a new plasma protein-precipitating agent that has the advantages of being cheap, efficient and suitable for drugs which are insoluble in common organic solvents. Owing to the short run time (1.2 min), rapid analysis of hundreds of plasma samples per day was possible, which makes the presented validated UPLC method suitable for the pharmacokinetic studies and biomedical analysis of EMPA.

## Additional file

**Additional file 1: Fig. S1.** Chromatograms obtained after using different pH values in mobile phase. **Fig. S2.** Chromatograms obtained after elution with mobile phase consists of; (acetonitrile: 0.1% trifluoroacetic acid, 20:80, v/v), b) (acetonitrile: 0.1% trifluoroacetic acid, 30:70, v/v), b) (acetonitrile: 0.1% trifluoroacetic acid, 40:60, v/v). **Fig. S3.** Chromatograms obtained after using different diluting solvents. **Fig. S4.** Chromatograms obtained after using different column temperatures.

## Data Availability

All data and materials are all provided.
