# Supplementary material for: A UPLC/DAD method for simultaneous determination of empagliflozin and three related substances in spiked human plasma
Source: BMC Chem. 2019 Jul 9;13(1):83. doi: 10.1186/s13065-019-0604-9 (PMC6661951; doi:10.1186/s13065-019-0604-9)
Supplement: Supplementary file 1 — Additional file 1: Fig. S1. Chromatograms obtained after using different pH values in mobile phase. Fig. S2. Chromatograms obtained after elution with mobile phase consists of; (acetonitrile: 0.1% trifluoroacetic acid, 20:80, v/v), b) (acetonitrile: 0.1% trifluoroacetic acid, 30:70, v/v), b) (acetonitrile: 0.1% trifluoroacetic acid, 40:60, v/v). Fig. S3. Chromatograms obtained after using different diluting solvents. Fig. S4. Chromatograms obtained after using different column temperatures. [file 13065_2019_604_MOESM1_ESM.docx]

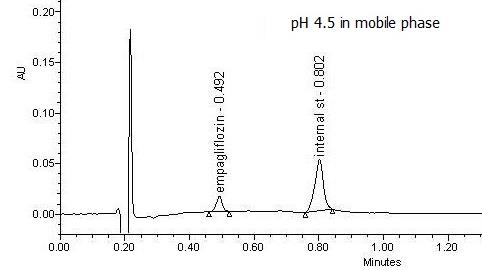


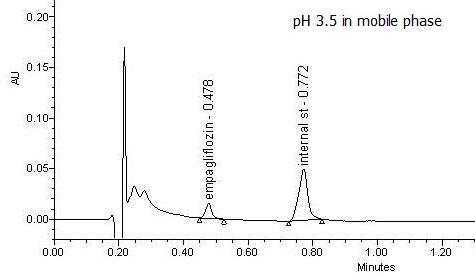


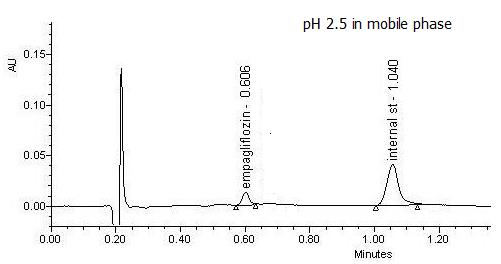


**Fig. S1.** Chromatograms obtained after using different pH values in mobile phase.


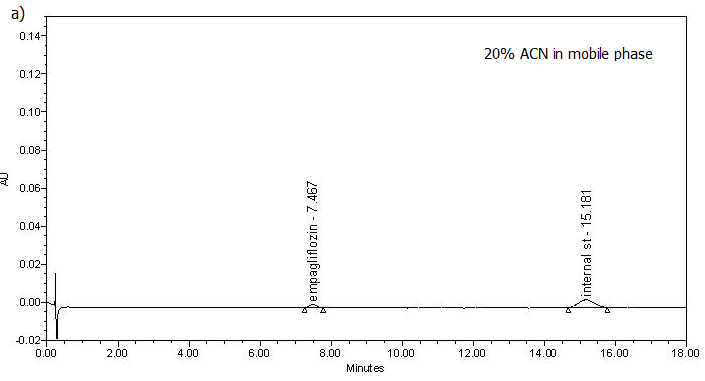


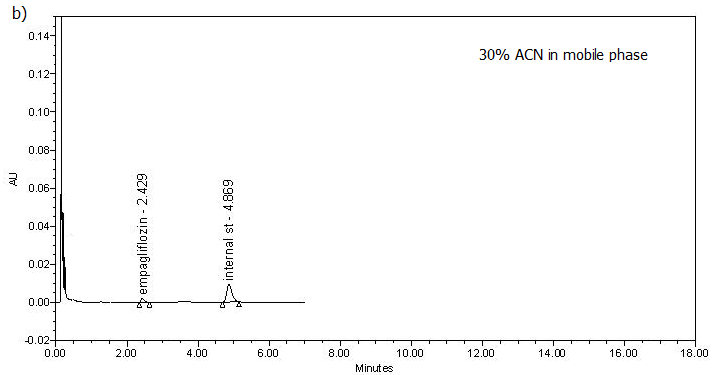


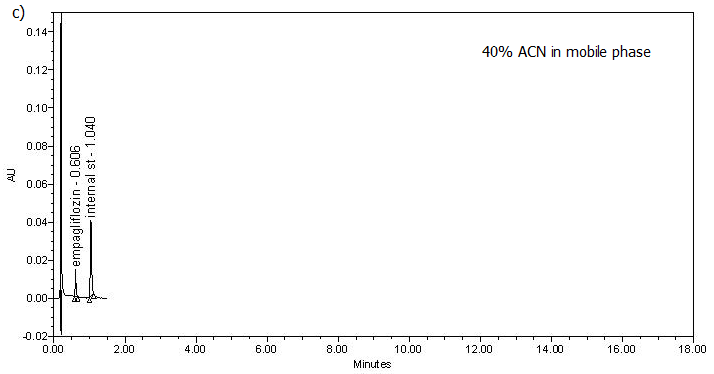


**Fig. S2.** Chromatograms obtained after elution with mobile phase consists of; (acetonitrile: 0.1% trifluoroacetic acid, 20:80, v/v), b) (acetonitrile: 0.1% trifluoroacetic acid, 30:70, v/v), b) (acetonitrile: 0.1% trifluoroacetic acid, 40:60, v/v).


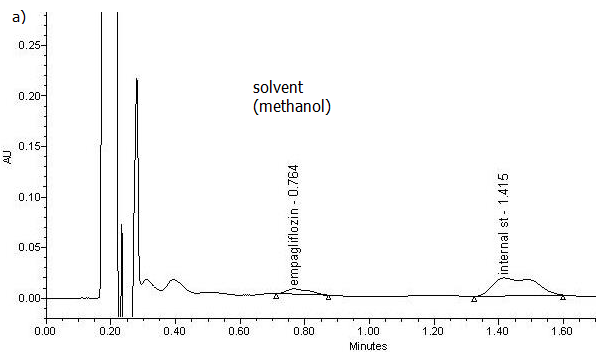


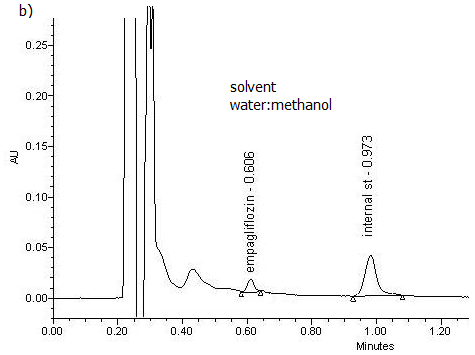


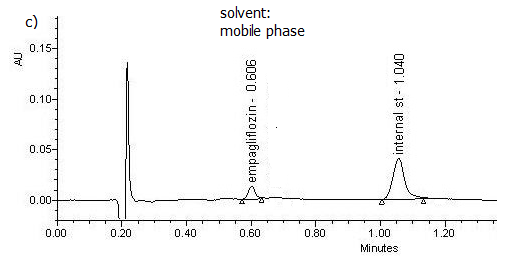


**Fig. S3.** Chromatograms obtained after using different diluting solvents.


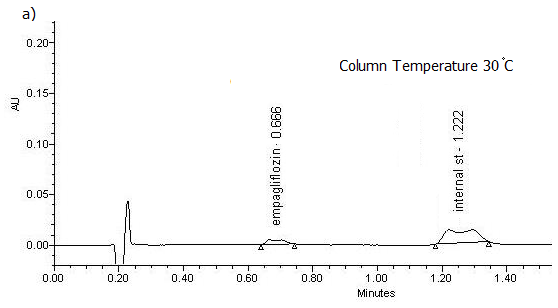


**
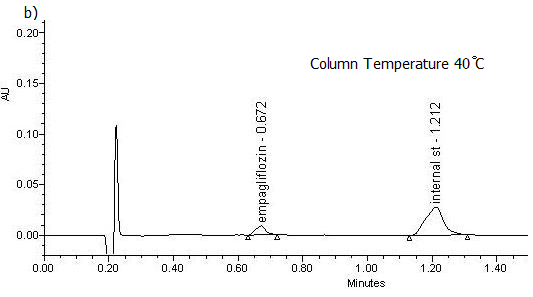
**

**
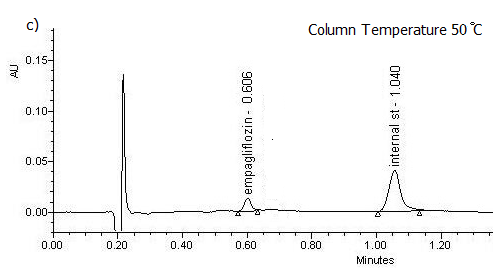
**

**Fig. S4.** Chromatograms obtained after using different column temperatures.
